# Enhanced protection conferred by mucosal BCG vaccination associates with presence of antigen-specific lung tissue-resident PD-1^+^ KLRG1^−^ CD4^+^ T cells

**DOI:** 10.1038/s41385-018-0109-1

**Published:** 2018-11-16

**Authors:** N. C. Bull, E. Stylianou, D. A. Kaveh, N. Pinpathomrat, J. Pasricha, R. Harrington-Kandt, M. C. Garcia-Pelayo, P. J. Hogarth, H. McShane

**Affiliations:** 1 The Jenner Institute, University of Oxford, Oxford, UK; 2 Vaccine Immunology Team, Department of Bacteriology, Animal & Plant Health Agency (APHA), Addlestone, Surrey UK

## Abstract

BCG, the only vaccine licensed against tuberculosis, demonstrates variable efficacy in humans. Recent preclinical studies highlight the potential for mucosal BCG vaccination to improve protection. Lung tissue-resident memory T cells reside within the parenchyma, potentially playing an important role in protective immunity to tuberculosis. We hypothesised that mucosal BCG vaccination may enhance generation of lung tissue-resident T cells, affording improved protection against *Mycobacterium tuberculosis*. In a mouse model, mucosal intranasal (IN) BCG vaccination conferred superior protection in the lungs compared to the systemic intradermal (ID) route. Intravascular staining allowed discrimination of lung tissue-resident CD4^+^ T cells from those in the lung vasculature, revealing that mucosal vaccination resulted in an increased frequency of antigen-specific tissue-resident CD4^+^ T cells compared to systemic vaccination. Tissue-resident CD4^+^ T cells induced by mucosal BCG displayed enhanced proliferative capacity compared to lung vascular and splenic CD4^+^ T cells. Only mucosal BCG induced antigen-specific tissue-resident T cells expressing a PD-1^+^ KLRG1^−^ cell-surface phenotype. These cells constitute a BCG-induced population which may be responsible for the enhanced protection observed with IN vaccination. We demonstrate that mucosal BCG vaccination significantly improves protection over systemic BCG and this correlates with a novel population of BCG-induced lung tissue-resident CD4^+^ T cells.

## Introduction

Tuberculosis (TB), resulting from infection with *Mycobacterium tuberculosis (M. tb)*, was responsible for 1.7 million deaths in 2016, making it the leading cause of death from infectious disease worldwide^1^.

Vaccination offers the most sustainable and cost-effective solution for long-term control of any infectious disease. The only vaccine currently available against TB is bacille Calmette-Guérin (BCG), a live attenuated strain of the cattle TB pathogen, *Mycobacterium bovis* (*M. bovis*). Despite providing some protection against disseminated disease in childhood, very limited protection has been reported against adult pulmonary disease^2^.

There is strong evidence that a T helper 1 (Th1)-polarised CD4^+^ T cell response plays a critical role in protection against *M. tb*^3–7^. However, studies demonstrate that the frequency of *M. tb*- or *M. bovis*-specific Th1 cells in the blood and lymphoid organs of humans, mice and cattle does not always correlate with protection^8–10^. Similarly, magnitude and frequency of vaccine-induced interferon gamma (IFN-γ) responses fail to predict immunity^8,10^. This lack of a robust correlate of protection presents a major hurdle to the development of improved TB vaccines. There is a real need for greater understanding of the properties of a protective host response, specifically the local response at the site of infection in the lung. This will facilitate the rational design of improved vaccines.

Recently, a new subset of memory T cells has been described, tissue-resident memory T cells. The defining feature of these cells is their persistence in non-lymphoid tissues and lack of re-circulation through the body. They have been shown to be present locally at the site of infection in multiple different tissues, have the ability to rapidly respond to pathogenic challenge in situ independently of T cell recruitment from the blood, and are able to coordinate recruitment of immune cells to tissue sites^11–17^. Development of an in vivo labelling technique to discriminate between cells present within the vasculature of an organ and those resident within the parenchyma has allowed definitive identification of tissue-resident cells^11,16,18,19^. This technique has now been utilised to identify a population of CD4^+^ T cells present within the lung parenchyma post-*M. tb* infection^20–22^. These cells are reported to mediate superior protection against *M. tb* infection and have also been demonstrated to rapidly migrate back to the lung following adoptive transfer^20^. Several studies characterise this parenchymal population as expressing reduced levels of KLRG1 and increased levels of PD-1 and CXCR3 (refs ^20,21,23^).

Exploiting development of lung tissue-resident memory T cells in the design of improved vaccines against TB is an exciting new prospect. To date, no data have been published on the induction of lung tissue-resident T cells following BCG vaccination utilising the intravascular staining technique. Woodworth et al.^24^ employed the technique to investigate responses to a subunit vaccine against TB. Immunisation generated polyfunctional CD4^+^ T cells which preferentially localised to the parenchyma of the lung and expressed reduced levels of KLRG1 on their cell surface; a phenotype associated with tissue-residence and enhanced control of bacterial growth^20,22,25^.

Mimicking the natural route of *M. tb* infection has been suggested as a possible means of improving the protective efficacy of vaccines^26^. Studies in several species (mice, guinea pigs and non-human primates) demonstrate that BCG vaccination by delivery to the lung mucosa is more protective against aerosol *M. tb* challenge than parenterally delivered BCG^27–32^. It is possible that delivery of BCG via mucosal routes has a direct effect on the local environment in the lung, specifically on the development of lung tissue-resident T cells. A recent study by Perdomo et al.^29^ linked mucosal delivery of BCG and generation of tissue-resident memory T cells in the lung, but these data were not achieved using intravascular staining. Previous studies using intravascular staining reveal that >95% of CD4^+^ T cells and >99% of total lymphocytes isolated from naïve murine lung via standard methods were in fact present in the vasculature of the lung rather than the parenchyma^19,20^. Therefore, it is important to evaluate tissue-resident responses utilising this technique in order to ensure that T cells truly present in the parenchyma are being analysed.

Here we demonstrate that delivering BCG via a mucosal route enhances protection against *M. tb* infection in the lung, and this protection is associated with induction of a significant population of antigen-specific lung tissue-resident CD4^+^ T cells. We refer to these cells as tissue-resident as they were identified within the lung parenchyma through intravascular staining. While this technique is extremely valuable for providing discrimination between cells present in the parenchyma and the vasculature, it does not allow us to make an assessment of the permanence of their state of residence. Thus, while we define this population as tissue-resident, we can only truly state that they were resident at the time intravascular staining was carried out.

## Results

### Mucosal BCG vaccination confers enhanced protection against *M. tb* infection

**Fig. 1:**
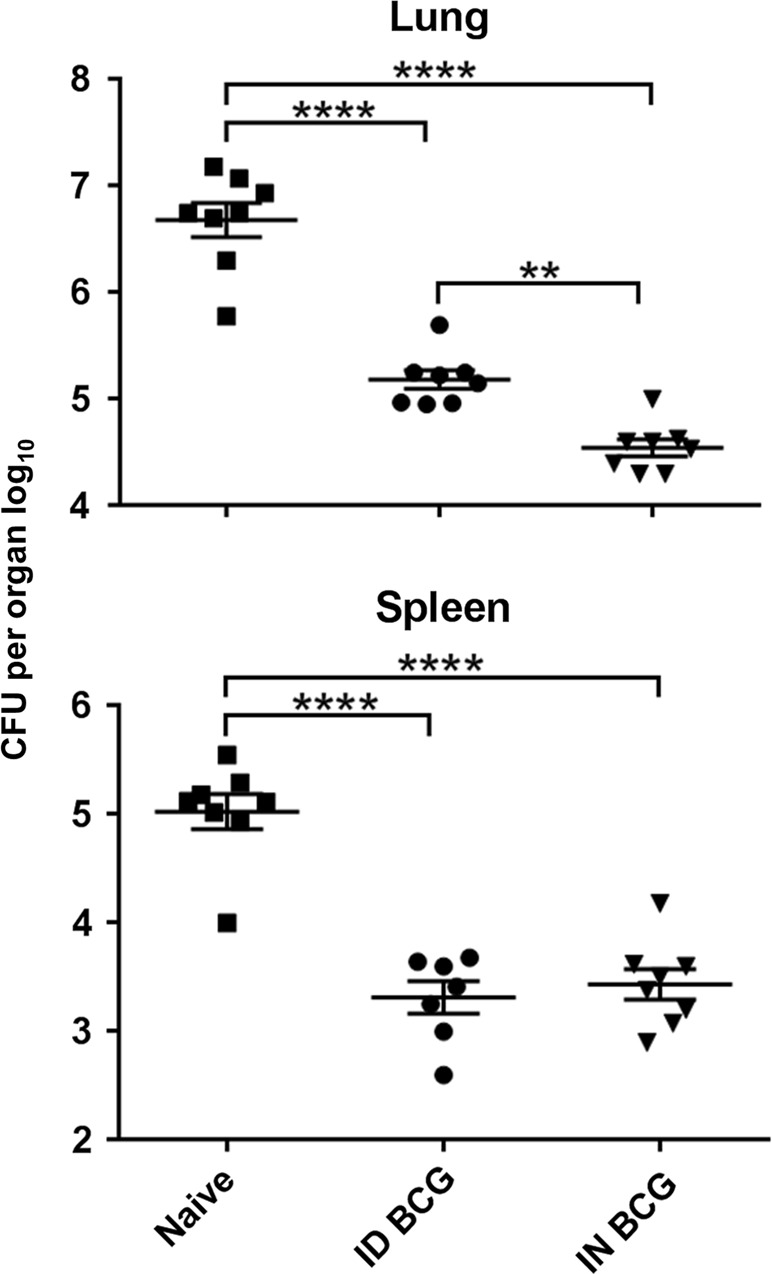
Mucosal BCG vaccination provides enhanced protection against *M. tb* infection in the lung. Mice immunised with BCG via IN or ID route were challenged 6 weeks later with *M. tb* via aerosol. Four weeks post-challenge, CFU were enumerated in lungs and spleen. Individual log_10_ CFU counts are shown with bars indicating mean ± standard error of the mean (SEM) (*n* ≥ 7). One-way ANOVA with Tukey’s post-test for significance; ****P < 0.0001; **P < 0.01. Data are representative of one of two independent experiments

Mucosal intranasal (IN) BCG vaccination conferred superior protection in the lungs of mice infected with aerosol *M. tb*, inducing a 0.7 log_10_ reduction in bacterial burden compared with mice receiving systemic intradermal (ID) BCG vaccination (*P* = 0.0022) (Fig. 1). There was no significant difference in bacterial load in the spleens of mice receiving BCG by either route.

### Mucosal BCG vaccination results in recruitment of CD4^+^ T cells to the lung parenchyma and increased frequency of antigen-specific CD4^+^ T cells in both the lung parenchyma and Broncho-alveolar lavage

**Fig. 2:**
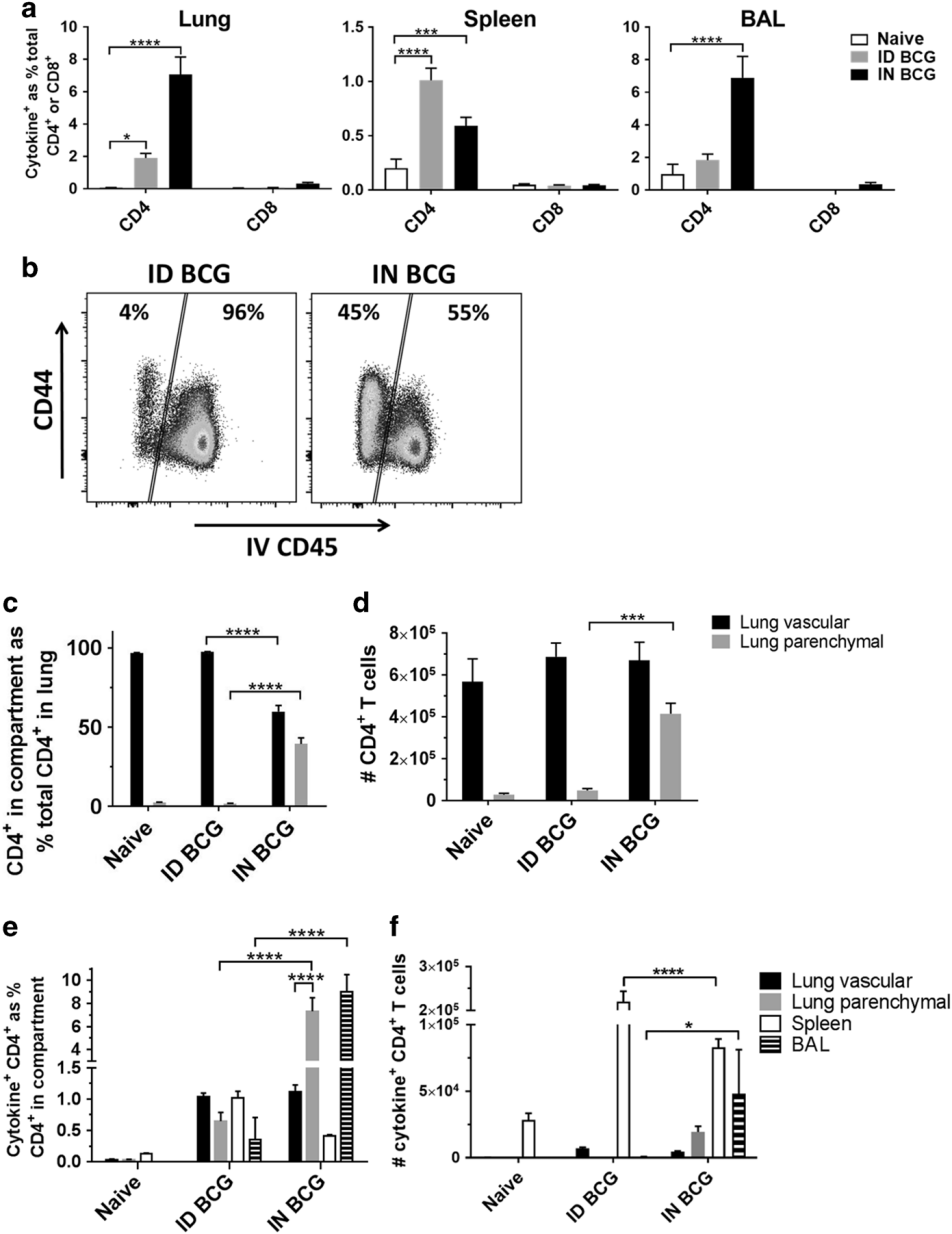
Mucosal BCG recruits CD4^+^ T cells to the lung parenchyma and induces greater frequency of antigen-specific CD4^+^ T cells in the lung parenchyma and BAL. Six weeks after immunisation with BCG via IN or ID route, intravascular staining and ICS identified populations of lung parenchymal and lung vascular antigen- (PPD-T)-specific (cytokine^+^) CD4^+^ or CD8^+^ T cells producing IFN-γ, TNF-α or IL-2 alone or in combination. **a** Frequency of BCG-induced antigen-specific cytokine^+^ CD4^+^ and CD8^+^ T cells in the lung, spleen and BAL. **b** Representative flow cytometry plots showing the proportion of lung parenchymal and lung vascular CD4^+^ T cells in the lungs of ID- or IN-immunised mice following intravascular anti-CD45 staining. **c** Frequency of lung parenchymal and lung vascular CD4^+^ T cells as a % of total CD4^+^ T cells isolated from the lung. **d** Number of CD4^+^ T cells in the lung parenchymal and lung vascular compartments. **e** Frequency of BCG-induced antigen-specific cytokine^+^ CD4^+^ T cells. **f** Number of BCG-induced antigen-specific cytokine^+^ CD4^+^ T cells. For **a**, **c**–**f** bars represent mean ± SEM (*n* = 6). Two-way ANOVA with Sidak’s post-test (**a**, **c**, **d**) or Tukey’s post-test (**e**, **f**); ****P < 0.0001, ***P < 0.001. Data are representative of one of two independent experiments

In vitro intracellular cytokine staining enabled identification of antigen-specific CD4^+^ and CD8^+^ T cells induced following BCG vaccination, through their production of IFN-γ, tumor necrosis factor-α (TNF-α) or interleukin-2 (IL-2) (cytokine^+^) in response to stimulation with cognate antigen (Fig. 2a). Both IN and ID BCG vaccination induced significant populations of antigen-specific CD4^+^ T cells in the lung and spleen, while IN BCG alone induced a significant population of antigen-specific CD4^+^ T cells in the broncho-alveolar lavage (BAL). There was no significant antigen-specific CD8^+^ T cell response present in any of the tissues investigated following either route of vaccination.

Intravascular anti-CD45 staining enabled further analysis of the CD4^+^ T cell population in the lung, allowing discrimination between cells present in the lung parenchyma and those present in the lung vasculature^19^ (full gating strategy in Supplementary Figure 1). IN BCG vaccination resulted in a significantly higher proportion of total CD4^+^ T cells in the lung being located within the parenchyma (39%) compared to ID BCG vaccination (2%) (*P* < 0.0001) (Fig. 2b, c). Analyses of cell counts confirmed these observations, with 4.1 × 10^5^ parenchymal CD4^+^ T cells after IN BCG compared to 0.5 × 10^5^ after ID BCG (*P* = 0.0009) (Fig. 2d). BCG vaccination by either route did not induce significant changes in the number of total CD4^+^ T cells in the lung vasculature.

IN vaccination induced a greater than 10-fold increase in frequency of antigen-specific CD4^+^ T cells in the lung parenchyma (7.3% vs 0.6%) and BAL (9.0% vs 0.4%), compared to ID vaccination (*P* < 0.0001) (Fig. 2e). This demonstrates an enrichment of antigen-specific CD4^+^ T cells locally within the tissue of the lung and the airways following mucosal BCG. There was no difference in frequency of antigen-specific CD4^+^ T cells in the lung vasculature between both groups. Analyses of cell counts follow a similar pattern, with an increased number of antigen-specific CD4^+^ T cells present in the lung parenchyma and BAL following IN rather than ID BCG (Fig. 2f). This increase only reached statistical significance for the BAL (4.2 × 10^2^ vs 4.8 × 10^4^, *P* = 0.011). There was no significant difference in number of antigen-specific CD4^+^ T cells in the lung vasculature between both groups. Boolean gating analysis reveals that the majority of antigen-specific CD4^+^ T cells generated by BCG vaccination in the lung parenchyma, lung vasculature, spleen and BAL are multifunctional (Supplementary Figure 2).

### Distinct phenotypic differences between antigen-specific CD4^+^ T cells induced following mucosal or systemic BCG vaccination

**Fig. 3:**
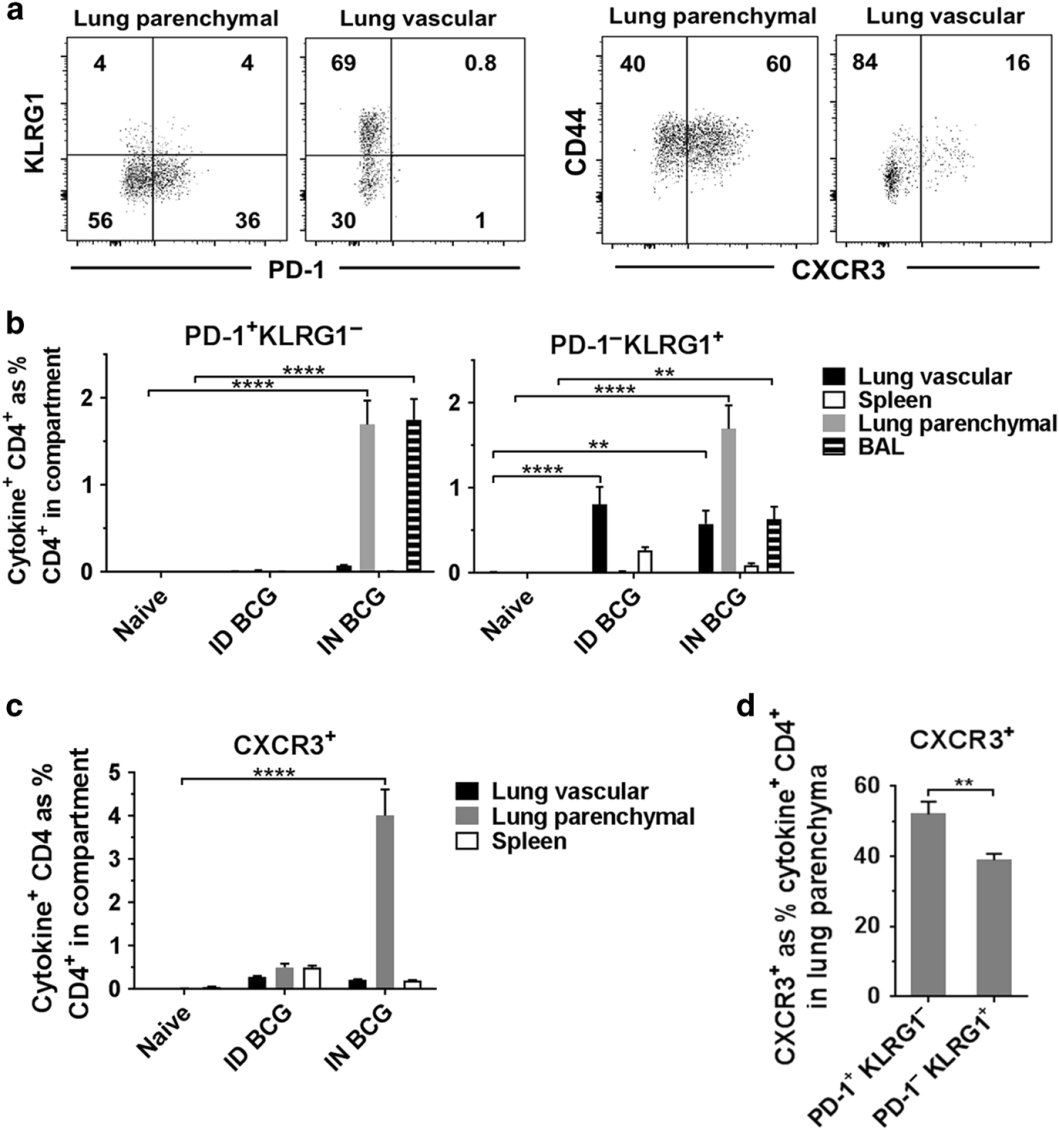
Mucosal, but not ID BCG, induces PD-1^+^ KLRG1^−^ CD4^+^ T cells in the lung parenchyma and BAL. Six weeks after immunisation with BCG via IN or ID route, intravascular staining and ICS identified antigen-specific (cytokine^+^) PD-1^+^ KLRG1^−^ and PD-1^−^ KLRG1^+^ CD4^+^ T cells. **a** Representative plots from IN-immunised mice, pre-gated on cytokine^+^ CD4^+^ T cells, showing surface staining for PD-1, KLRG1 and CXCR3 in the lung parenchyma and lung vasculature. **b** Frequency of cytokine^+^ CD4^+^ T cells expressing a PD-1^+^ KLRG1^−^ or PD-1^−^ KLRG1^+^ phenotype. **c** Frequency of cytokine^+^ CD4^+^ T cells expressing CXCR3. **d** Proportion of lung parenchymal cytokine^+^ CD4^+^ T cells expressing CXCR3 in PD-1/KLRG1 subsets in mice after mucosal BCG vaccination. For **b**–**d** bars represent mean ± SEM (*n* = 6). Two-way ANOVA with Tukey’s post-test (**b**) or Sidak’s post-test (**c**); ****P < 0.0001, ***P < 0.001, **P < 0.01. Unpaired *t*-test (**d**); **P < 0.01. Data are representative of one of two independent experiments

In order to further characterise the antigen-specific CD4^+^ T cells induced locally in the lung and peripherally in the spleen following IN and ID BCG vaccination, we investigated expression of the cell-surface markers PD-1, KLRG1 and CXCR3 (Fig. 3a), which have been previously described as markers able to define different subsets of CD4^+^ T cells present during *M. tb* infection. We report that antigen-specific CD4^+^ T cells expressing a PD-1^+^ KLRG1^−^ phenotype were exclusively present within the lung parenchyma and BAL following IN BCG vaccination (*P* < 0.0001) and were not induced following ID BCG (Fig. 3b). Antigen-specific CD4^+^ T cells expressing the opposing PD-1^−^ KLRG1^+^ phenotype were present in the lung vasculature, lung parenchyma and BAL following IN BCG and in the lung vasculature following ID BCG. We also investigated expression of CXCR3 as this has been described as representing a population of CD4^+^ T cells capable of localising to the lung parenchyma during *M. tb* infection^20^. We observed that antigen-specific CXCR3^+^ CD4^+^ T cells were only present following IN BCG and found only in the lung parenchyma (*P* < 0.0001) (Fig. 3c). We investigated whether there was any relationship between expression of PD-1, KLRG1 and CXCR3 on lung parenchymal antigen-specific CD4^+^ T cells following IN BCG. While CXCR3 is expressed on cells with both a PD-1^+^ KLRG1^−^ and PD-1^−^ KLRG1^+^ phenotype, there was a significantly greater frequency of CXCR3^+^ cells within the PD-1^+^ KLRG1^−^ population, 52% compared to 39% (*P* = 0.0029) (Fig. 3d).

### Enhanced proliferative capacity of lung parenchymal CD4^+^ T cells induced following mucosal BCG vaccination

**Fig. 4:**
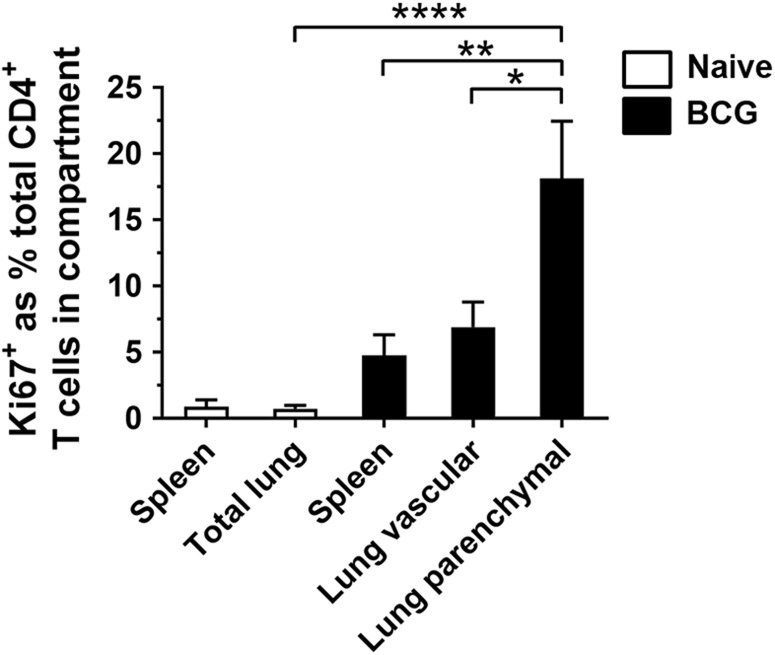
Lung parenchymal CD4^+^ T cells have greater proliferative capacity than lung vascular or splenic CD4^+^ T cells following mucosal BCG. Mice were vaccinated with BCG IN 6 weeks prior to intravascular staining. Purified populations of lung parenchymal, lung vascular and splenic CD4^+^ T cells were obtained by sorting. Naïve CD4^+^ lung and spleen T cells were sorted without intravascular staining. Cells were cultured for 3 days with PPD-T before proliferating cells were identified with Ki67 staining. To account for non-specific expression of Ki67, unstimulated values were subtracted from stimulated. Graph shows frequency of Ki67^+^ CD4^+^ T cells in the total lung and spleen of naïve mice and the lung parenchyma, lung vasculature and spleen of BCG-immunised mice. Bars represent mean ± SEM (*n* = 6). One-way ANOVA with Tukey’s post-test, ****P < 0.0001; **Ρ < 0.01, *Ρ < 0.05. Data are pooled from three independent experiments

We investigated the proliferative capacity of CD4^+^ T cells in the lung and spleen of mice following IN BCG vaccination through analysis of expression of Ki67, a well-established marker of proliferation expressed during active phases of the cell cycle^33–37^. CD4^+^ T cells were isolated from the lung parenchyma (98.9% purity), lung vasculature (99.8% purity) and spleen (99.6% purity) of immunised mice through flow-cytometric cell sorting and stimulated with antigen for 72 h. A significantly greater frequency of Ki67^+^ CD4^+^ T cells was present in the lung parenchyma of immunised mice compared to the lungs of naïve mice (*P* < 0.0001) (Fig. 4). The highest frequency of BCG-induced Ki67^+^ CD4^+^ T cells was detected in the lung parenchyma (18%) compared to the lung vasculature (7%) and spleen (5%) (*P* = 0.0118 and *P* = 0.0022, respectively).

### Durability of responses to mucosal BCG

**Fig. 5:**
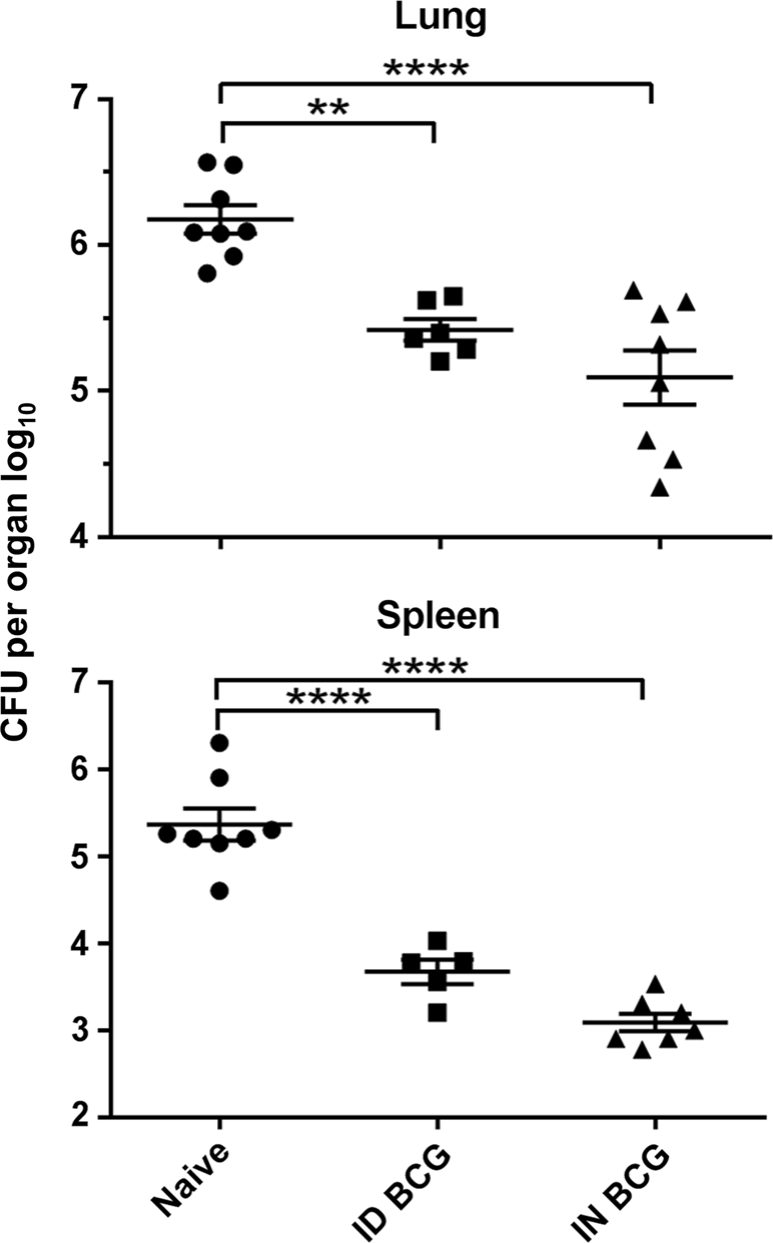
BCG-immunised mice are protected against *M. tb* infection 26 weeks post-immunisation. IN or ID BCG-immunised mice were challenged 26 weeks later with *M. tb* via aerosol. Four weeks post-challenge, CFU were enumerated in lung and spleens. Individual log_10_ CFU values are shown with bars indicating mean ± SEM (*n* ≥ 5). One-way ANOVA with Tukey’s post-test for significance; ****P < 0.0001; **P < 0.01

In order to investigate whether the route of BCG delivery affected the durability of the protective responses, IN or ID vaccinated mice were challenged with *M. tb* 26 weeks post-vaccination. Both vaccinated groups had significantly lower bacterial burdens in their lungs (IN 1.1 vs ID 0.8 log_10_ protection) and spleens (IN 2.3 vs ID 1.7 log_10_ protection) compared to the control group, and although there was a trend towards improved protection with IN vs ID BCG, the difference did not reach statistical significance (*P* = 0.26) (Fig. 5).

**Fig. 6:**
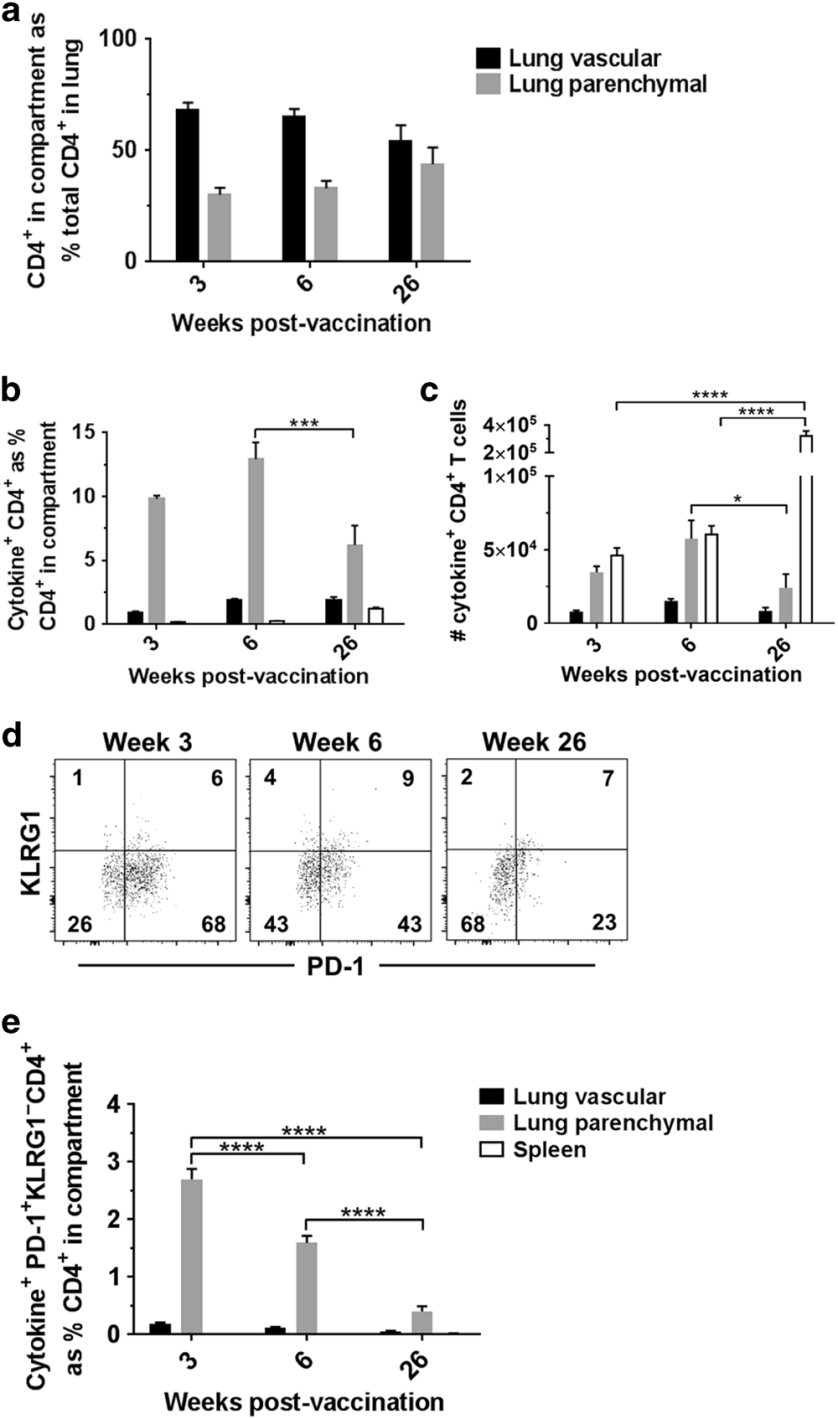
Lung parenchymal PD-1^+^ KLRG1^−^ CD4^+^ T cells decrease with time after mucosal BCG vaccination. Mice were vaccinated with BCG via IN or ID route 3, 6 or 26 weeks prior to intravascular staining and ICS to identify populations of lung parenchymal, lung vascular and spleen antigen-specific (cytokine^+^) CD4^+^ T cells. **a** Frequency of lung parenchymal and lung vascular CD4^+^ T cells as a % of total CD4^+^ T cells isolated from the lung. Statistical comparison conducted between the same compartments at different time points, all non-significant. **b** Frequency of BCG-induced antigen-specific cytokine^+^ CD4^+^ T cells. **c** Number of BCG-induced antigen-specific cytokine^+^ CD4^+^ T cells. **d** Representative plots from IN-immunised mice, pre-gated on cytokine^+^ CD4^+^ T cells, showing surface staining for PD-1 and KLRG1 in the lung parenchyma. **e** Frequency of cytokine^+^ CD4^+^ T cells expressing a PD-1^+^ KLRG1^−^ phenotype. For all graphs, bars represent mean ± SEM (*n* = 6). Two-way ANOVA with Sidak’s post-test, ***Ρ < 0.001, ****Ρ < 0.0001

To investigate whether this reduction in efficacy of IN BCG was due to a decrease in the frequency of lung parenchymal CD4^+^ T cells, we measured immunological responses at 3, 6 and 26 weeks post-IN BCG vaccination. We found that while the frequency of total CD4^+^ T cells in both lung compartments remains stable up to 26 weeks following vaccination, with no significant differences when comparing the same compartment across different time points (Fig. 6a), the frequency of antigen-specific CD4^+^ T cells in the lung parenchyma is significantly lower at 26 weeks post-vaccination (6%) than at 6 weeks post-vaccination (13%) (*P* = 0.0007) (Fig. 6b). Analyses of cell counts also shows a decrease in the number of antigen-specific CD4^+^ T cells in the lung parenchyma from week 6 (5.7 × 10^4^) to week 26 (2.4 × 10^4^) (*P* = 0.0445) (Fig. 6c). In contrast, the number of antigen-specific CD4^+^ T cells present in the spleen was higher at week 26 post-vaccination (3.2 × 10^5^) compared to both week 3 (4.7 × 10^4^) and week 6 (6.1 × 10^4^) (*P* < 0.0001).

Additionally, expression of a PD-1^+^ KLRG1^−^ phenotype on antigen-specific CD4^+^ T cells in the parenchyma was investigated (Fig. 6d). The frequency of phenotype expression was significantly lower (0.4%) at week 26 post-vaccination than at either week 3 (2.7%) or 6 (1.6%) post-vaccination (*P* < 0.0001) (Fig. 6e).

## Discussion

Consistent with recent reports^29,30^, mucosal (IN) BCG vaccination conferred superior protection in the lungs of mice infected with *M. tb*, compared to mice receiving systemic (ID) BCG vaccination. However, protection in the spleens of mice receiving BCG was equal, suggesting that mucosal BCG induces enhanced locally protective mechanisms within the lungs, but not additional protection against systemic dissemination of mycobacteria.

While the mechanism for this additional local protection has yet to be determined, we demonstrate that delivery of BCG mucosally recruits CD4^+^ T cells from the periphery to the lung parenchyma, increasing the total number of cells present at the primary site of infection. Additionally, within this population, there is an increased frequency of antigen-specific CD4^+^ T cells. Impairment of dendritic cell (DC)-mediated antigen presentation in the lung and delay of DC migration to the draining lymph nodes are mechanisms of immune evasion employed by *M. tb* upon entry to the lungs^38^. This interference with initiation of effective adaptive immune responses allows the bacteria to expand within the lung before antigen-specific T cells accumulate sufficiently to inhibit bacterial growth^39^. The increased influx of antigen-specific CD4^+^ T cells into the parenchyma following mucosal BCG vaccination may be responsible for the enhanced protection compared to systemic BCG observed here. Although a causal connection cannot be definitively demonstrated through the experiments described here, we hypothesise that an increased number of antigen-specific cells, situated at the site of infection and primed to respond to mycobacteria, may help to control this early phase of *M. tb* growth. Further work is needed to determine the precise mechanisms of immune protection involved, as these are not elucidated within this study. Experiments comparing acute inflammatory responses following *M. tb* challenge in mucosally and systemically vaccinated animals would provide valuable mechanistic insight.

We did not observe an antigen-specific CD8^+^ T cell response in the lungs, spleen or BAL following either mucosal or systemic BCG vaccination. This is consistent with previous reports demonstrating evidence for a role for CD8^+^ T cells in novel vaccine regimens including prime-boost regimes and recombinant BCG vaccine candidates, rather than following BCG alone^40–42^.

It is unclear why the additional protection observed in the lung does not also manifest in a reduced burden of *M. tb* in the spleen. This finding may suggest that extrapulmonary dissemination of mycobacteria occurs prior to enhanced local control by lung tissue-resident CD4^+^ T cells taking effect, although further work is required to understand the precise timing of these events during the course of infection. It is currently unclear whether this has any implications for the efficacy of mucosal vaccination against pulmonary TB, compared to extrapulmonary disease.

While intravascular staining enabled us to identify antigen-specific CD4^+^ T cells present in the lung parenchyma, we cannot definitively state whether these cells are permanently resident within the lung or are in fact circulating and in the process of transiting through the parenchyma. Connor et al.^43^ utilised FTY720 treatment to prevent lymphocyte egress from lymph nodes and demonstrated that if this treatment was administered at the time of BCG vaccination, protection against subsequent infection was reduced. However, administering treatment during infectious challenge to mice previously vaccinated with BCG did not impact on protection. This suggests that BCG-mediated protection is dependent on initial migration of lymphocytes to the lung from the lymph nodes during vaccine-induced priming and subsequent retention of these cells in the lung. This is in line with our finding that CD4^+^ T cells are recruited from the periphery to the parenchyma following mucosal BCG vaccination.

Mucosal, but not systemic BCG, induced antigen-specific CD4^+^ T cells in the broncho-alveolar space. In agreement with this finding, several studies have demonstrated that TB vaccines delivered to the lung mucosa are able to elicit a T cell response within the airway lumen and restrict replication of *M. tb* in the first week following infection^44–51^. The data presented here, combined with these reports, indicate that airway luminal T cells may be able to provide a first line of defence against *M. tb* infection and should be considered as a target population when designing new vaccination regimes^49^.

BCG also produces numerous innate effects, which may have contributed to the enhanced protection observed following mucosal vaccination^52^. Additionally, mucosal delivery of BCG has been shown to induce granulomatous infiltration, which may influence lung homing of T cells following subsequent challenge, as well as create specific niches for accumulation of tissue-resident T cells^53,54^. Further work is required to determine the extent to which these factors contribute to the improvement in protection seen with mucosal BCG. This will require investigation in non-murine models to account for observed differences in granulomatous responses between mice and humans^55^.

In order to further characterise the antigen-specific CD4^+^ T cells induced locally in the lung and airways, we investigated expression of the cell-surface markers PD-1, KLRG1 and CXCR3. These have been previously described as markers defining functional subsets of CD4^+^ T cells present during *M. tb* infection. PD-1^+^ CD4^+^ T cells represent less differentiated cells, exhibiting greater proliferative and protective capacity than PD-1^−^ cells^21,23^. Expression of KLRG1 is associated with terminal differentiation^23,56^ and inability to migrate into the lung parenchyma and protect against *M. tb* infection^20,22^. Moguche et al.^57^ recently reported that chronic antigenic stimulation during *M. tb* infection promotes the maintenance of KLRG1^+^ CD4^+^ T cells, which they describe as terminally differentiated, functionally exhausted cells. CXCR3 expression is considered to identify CD4^+^ T cells capable of localising to the lung parenchyma during *M. tb* infection^20^.

These markers have been investigated in the context of TB vaccination and *M. tb* infection^24,25,29,44^, but no data are published on their expression following mucosal delivery of BCG, utilising the intravascular staining technique to definitively identify tissue-resident cells.

In this study, we report that an antigen-specific CD4^+^ T cell population with PD-1^+^ KLRG1^−^ cell-surface expression, associated with enhanced protection against *M. tb* infection, was only induced following mucosal BCG vaccination and was exclusively present within the lung parenchyma and airways. This suggests that delivery of antigen mucosally is critical for development of cells expressing this phenotypic combination. We also report that CXCR3 expression was present only on lung parenchymal CD4^+^ T cells following mucosal BCG vaccination, and that expression of this marker was highest on PD-1^+^ KLRG1^−^ lung parenchymal cells. This contrasts with recent studies^24,44^ which describe induction of antigen-specific T cells expressing CXCR3 in the lung vasculature. However, these studies used systemic immunisation with a subunit or viral-vectored TB vaccine rather than BCG, and are therefore not directly comparable. Further investigation into the induction of cells expressing this parenchyma-localising phenotype is required, as it may prove important for future vaccine design.

Additional phenotypic markers have been putatively associated with tissue-resident T cells, particularly CD69 and CD103. CD69 has been suggested to play a role in their maintenance within tissues, due to its ability to inhibit sphingosine-1-phosphate receptor 1 (S1PR1)-mediated T cell exit from secondary lymphoid organs. However, despite a number of papers using CD69 to identify tissue-resident memory T cells, Beura et al.^58^ have recently demonstrated it to be insufficient for determining stable residence in organs. Similarly, CD103 is only expressed by certain subsets of tissue-resident CD8^+^ T cells and not significantly by tissue-resident CD4^+^ T cells^16,59–61^. Currently, no definitive phenotype for lung tissue-resident CD4^+^ T cells exists and this is an area of ongoing study^62^.

We have also demonstrated that CD4^+^ T cells present within the lung parenchyma following mucosal BCG vaccination exhibit enhanced proliferative capacity compared to CD4^+^ T cells present within the lung vasculature or spleen. The ability to proliferate suggests that these cells are memory, rather than terminally differentiated effector T cells^63^. This is consistent with our data describing the presence of antigen-specific CD4^+^ T cells with a PD-1^+^ KLRG1^−^ phenotype in the lung parenchyma, associated with non-terminal differentiation^20,21,23,25^. The finding that lung parenchymal CD4^+^ T cells appear to have greater memory potential may explain the association between greater numbers of lung parenchymal CD4^+^ T cells and enhanced protection in the lung. It suggests that mucosal BCG is able to induce a population of CD4^+^ T cells within the lung tissue able to respond to pathogenic challenge in situ.

Protection against *M. tb* infection was also assessed 26 weeks post-vaccination, in order to determine whether both routes of administration provided equally durable protection. Both displayed significant protection compared to controls, and although there was a trend towards enhanced protection with mucosal BCG, it was no longer statistically significant. This may be due to the reduced frequency of antigen-specific PD-1^+^ KLRG1^−^ CD4^+^ T cells in the lung parenchyma. Further work will identify whether maintenance of higher frequencies of these cells would result in more durable enhanced protection.

There is incomplete understanding of the factors involved in the maintenance of lung tissue-resident memory T cells, and there are no data published on their longevity following TB vaccination utilising the intravascular staining technique. Longitudinal studies of CD8^+^ lung tissue-resident memory T cells show the population waning over time following clearance of influenza virus infection. Slutter et al.^64^ suggest that there is a requirement for continual replenishment of this population from the circulating memory T cell pool. However, Turner et al.^65^ used a murine model of allergic airway disease to demonstrate that only CD4^+^, not CD8^+^ tissue-resident T cells, persisted long term in the lung, following cessation of exposure to allergen. This suggests that there are differences in the dynamics of lung tissue-resident CD4^+^ and CD8^+^ T cell responses, which require further investigation.

It is also possible that maintenance of the tissue-resident population within the lung is reliant on the presence of live BCG bacilli, which have been shown to persist for up to 22 weeks post-vaccination^66^. Kaveh et al.^66^ used chemotherapy to clear BCG from vaccinated mice and demonstrated that in the absence of live bacilli, there was no longer a detectable antigen-specific CD4^+^ T cell response. This reliance on persistence of BCG for maintenance of a CD4^+^ T cell response may explain the findings of an earlier study by Palendira et al.^67^ The authors found no difference in protective efficacy of BCG between mice vaccinated mucosally and systemically, when antibiotics were administered to clear BCG between vaccination and subsequent *M. tb* challenge. It is highly likely that BCG bacilli were present in the lungs of mice in our study at both 6 and 26 weeks post-vaccination. This is a confounding factor when characterising lung tissue-resident T cells phenotypically in the context of BCG vaccination, which is why we have utilised intravascular staining as an additional means of identifying these cells by their location within the parenchyma.

In conclusion, we report that mucosal delivery of BCG provides enhanced protection in the lungs of mice challenged with *M. tb*, compared to systemic BCG immunisation. This was associated with increased numbers of CD4^+^ T cells in the lung parenchyma, a greater frequency of antigen-specific CD4^+^ T cells within this population and the presence of antigen-specific CD4^+^ T cells in the broncho-alveolar space. A proportion of these cells in the parenchyma and airways expressed a PD-1^+^ KLRG1^−^ CXCR3^+^ cell-surface phenotype. We propose that mucosal BCG vaccination induces specific local protective effects in the lung, and identify lung tissue-resident CD4^+^ cells as a key component of this response. An increased understanding of protective mechanisms induced by mucosal immunisation will inform rational vaccine development and new strategies to improve durability of protective efficacy.

## Methods

### Animals

Female specific-pathogen-free (SPF) BALB/c mice were supplied by Charles River UK Ltd (Margate, UK) or Envigo RMS UK Ltd (Huntingdon, UK) and used at 8 weeks of age. For Ki67 analysis, splenic antigen-presenting cells (APC) for co-culture were derived from a CD90.1 congenic BALB/c colony maintained at APHA (Weybridge, UK). Animals were housed in appropriate biological containment facilities, according to the Code of Practice for the Housing and Care of Animals Bred, Supplied or Used for Scientific Purposes. All animals were randomly assigned to treatment groups, housed in groups of 4–6 in individually ventilated cages and provided water and food *ad libitum*. After challenge with *M. tb*, all mice were assessed for clinical signs of TB daily. Provision of normally distributed data for immunological analyses required minimum sample size *n* = 6 (Kolmogorov and Smirnov test).

### Immunisation and aerosol *M. tb* challenge

Mice were immunised with the human vaccine strain *M. bovis* BCG Danish 1331 grown in-house in Middlebrook 7H9 broth (BD Biosciences, San Jose, CA, USA). A single dose of 2 × 10^5^ colony-forming units (CFU) of BCG in 50 µl inoculum was administered via the ID or IN route, both under brief general anaesthesia with isoflurane (Zoetis, London, UK). For ID immunisation, inoculum was injected in the pinnae of right and left ears, 25 µl in each; for IN immunisation, inoculum was administered equally to both nares. Mice were challenged using a Biaera AeroMP®-controlled nebuliser (Biaera Technologies, Hagerstown, MD, USA) contained in a Biosafety level 3 TCOL isolator (Total Containment Oxford Ltd, Bicester, UK). Animals were held in nose-only restrainers and exposed to aerosolised *M. tb* Erdman K01 (TMC107) (BEI Resources, Manassas, VA, USA), prepared at 1 × 10^6^ CFU/ml in the nebuliser. The programme was run for 10 min followed by a 5 min purge, airflow 12 l/min, and pressure 20 psig. Mice were infected with 50–100 CFU, verified 24 h after challenge in two mice per experiment. Four weeks post-challenge, mice were euthanased and lungs and spleens homogenised in 1 ml PBS using a Precellys 24 (Stretton Scientific, Stretton, UK). Homogenates were serially diluted in phosphate-buffered saline (PBS) (Sigma-Aldrich, St. Louis, MO, USA), plated in duplicate on modified 7H11 agar plates^68^ (APHA), and incubated for 4 weeks at 37 °C before counting colonies.

### Intravascular stain

Intravascular staining was performed using an amendment of the method described by Anderson et al.^19^ Briefly, 100 µl of PE-conjugated anti-CD45 monoclonal antibody (eBioscience, San Diego, CA, USA) at 0.75 µg/ml in PBS was administered via the lateral tail vein 1 min prior to euthanasia, to allow flow-cytometric discrimination between lung vascular cells (accessible to the stain) and lung parenchymal cells (inaccessible to the stain).

### Lymphocyte isolation

Spleen cells were isolated by passage through a 40 µm cell strainer, washed at 300 *g* for 8 min and re-suspended at 1 × 10^7^ cells/ml in Dulbecco’s Modified Eagle Media (DMEM) (Sigma-Aldrich) supplemented with fetal calf serum and penicillin/streptomycin (Thermo Fisher Scientific, Waltham, MA, USA) for assays.

Lung cells were isolated through use of a GentleMACS™ tissue dissociator using C tubes (Miltenyi Biotec, Bisley, UK). Cells were agitated at 37 °C for 1 h in supplemented DMEM with DNAse II (Sigma-Aldrich) and collagenase type I (Thermo Fisher Scientific), passed through a 40 µm cell strainer, washed and re-suspended at 5 × 10^6^ cells/ml in supplemented DMEM for assays.

BAL cells were isolated through in situ catheterisation of the trachea and flushing of the airways twice with individual volumes of 0.5 ml phosphate-buffered saline (PBS) (Sigma-Aldrich), which were subsequently pooled. Cells were washed and re-suspended in supplemented DMEM for assays.

### Flow cytometry

Cells isolated from spleen, lungs or BAL were cultured with 10 µg/ml Purified Protein Derivative of *M. tb* (PPD-T) (Statens Serum Institut, Copenhagen, DK), 1 µg/ml anti-CD28 (BD Biosciences) and 10 µg/ml Brefeldin A (Sigma-Aldrich) for 16 h at 37 °C/5% CO_2_. Cells were washed (300 *g*/5 min) and surface stained for 15 min/4 °C with pre-titrated antibodies: PD-1^−^FITC, CD44-BV785, CD8-AF700, KLRG1-PerCP-Cy5.5, CXCR3-BV421, live/dead-Zombie Aqua (all BioLegend, San Diego, CA, USA), CD90.2-eFluor 450 (eBioscience), and CD4-APC-H7 (BD Biosciences). Cells were then washed, treated with BD Biosciences Cytofix/Cytoperm as per the manufacturer’s instructions and stained intracellularly for 30 min/4 °C with IFN-γ-PE-Cy7, IL-2-APC (both eBioscience) and TNF-α-BV605 (BioLegend). Cells were washed again and acquired using an LSRFortessa™ analyser, utilising a 532 nm laser for PE and PE-conjugate excitation, with FACSDiva™ software (BD Biosciences). Final analysis was performed using FlowJo® software (Tree Star, Ashland, OR, USA) on a minimum of 100,000 live lymphocytes (50,000 for BAL).

### Ki67 expression

Purified CD4^+^ T cell populations were obtained from spleen and lung using flow-cytometric cell sorting on a MoFlo® Astrios™ with Summit acquisition software (Beckman Coulter, Brea, CA, USA). Cells were cultured with and without PPD-T at 10 µg/ml with the addition of 1 µg/ml anti-CD28 for 3 days at 37 °C/5% CO_2_, in co-culture with adherent spleen-derived APC from naïve congenic CD90.1 BALB/c mice. Briefly, spleens were processed as previously and incubated for 4 h/37 °C before removal of non-adherent cells by three aspirations of supplemented DMEM. Antigen and purified CD4^+^ T cells were immediately added to the culture. Any congenic T cells which had failed to be removed from the splenic APC co-culture were excluded from final flow-cytometric analysis through expression of CD90.1. Cell-surface staining was performed as previously with CD90.1-BV421, CD44-BV785, live/dead-Zombie Aqua (all BioLegend) and CD4-APC-H7 (BD Biosciences). After surface staining, cells were washed twice as previously and cold 70% ethanol added to the cell pellet while vortexing. Cells were incubated for 1 h/−20 °C, washed three times, stained for 30 min/room temperature with Ki67-PE (BioLegend) and washed twice before analysis, as previously.

### Statistical analysis

All data were analysed using GraphPad Prism 7 statistical package (GraphPad Software, La Jolla, CA, USA). When comparing two groups, an unpaired Student’s two-tailed *t*-test was performed. With three or more treatment groups the data were analysed by one-way ANOVA with appropriate multiple comparisons test as stated. Where two independent variables were compared, data were analysed by two-way ANOVA with appropriate multiple comparisons test as stated. Mycobacterial counts were log_10_ transformed before the statistical analysis. For all data, * represents *P* < 0.05, ** represents *Ρ* < 0.01, *** represents *Ρ* < 0.001 and **** represents *Ρ* < 0.0001.

## Electronic supplementary material

Supplementary Figure 1

Supplementary Figure 2
