# Supplementary material for: Enhanced protection conferred by mucosal BCG vaccination associates with presence of antigen-specific lung tissue-resident PD-1+ KLRG1− CD4+ T cells
Source: Mucosal Immunol. 2018 Nov 16;12(2):555–64. doi: 10.1038/s41385-018-0109-1 (PMC7051908; doi:10.1038/s41385-018-0109-1)
Supplement: Supplementary file 1 — Supplementary Figure 1 [file 41385_2018_109_MOESM1_ESM.pdf]

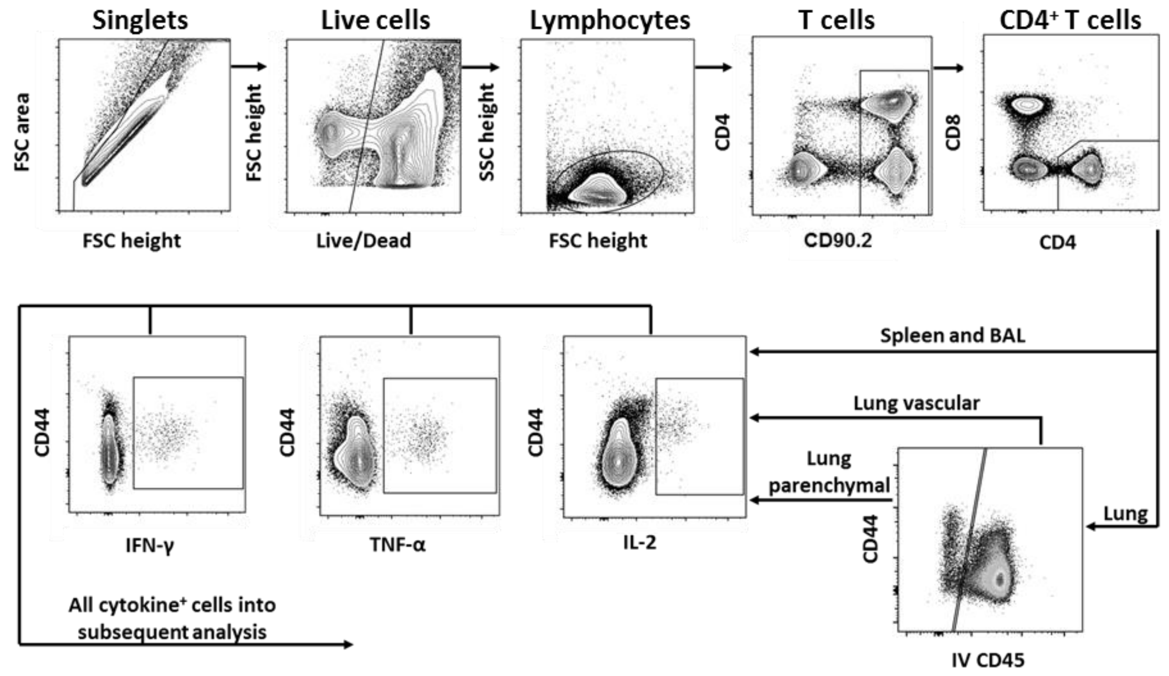

Supplementary Figure 1 Gating strategy for identification of cytokine^{+} CD4^{+} T cells. Cells are gated on singlets followed by live cells and then lymphocytes. T cells are identified as CD90.2^{+} before gating on CD4^{+} and identifying CD44^{hi} cells producing IFN-γ, TNF-α or IL-2 (cytokine^{+}). Boolean gating is then used to identify all CD4^{+} cells producing one or more of these cytokines. For analysis of lung lymphocytes, an additional step of gating based on intravascular stain is performed prior to identification of cytokine^{+} cells.
