# Supplementary material for: Enhanced protection conferred by mucosal BCG vaccination associates with presence of antigen-specific lung tissue-resident PD-1+ KLRG1− CD4+ T cells
Source: Mucosal Immunol. 2018 Nov 16;12(2):555–64. doi: 10.1038/s41385-018-0109-1 (PMC7051908; doi:10.1038/s41385-018-0109-1)
Supplement: Supplementary file 2 — Supplementary Figure 2 [file 41385_2018_109_MOESM2_ESM.pdf]

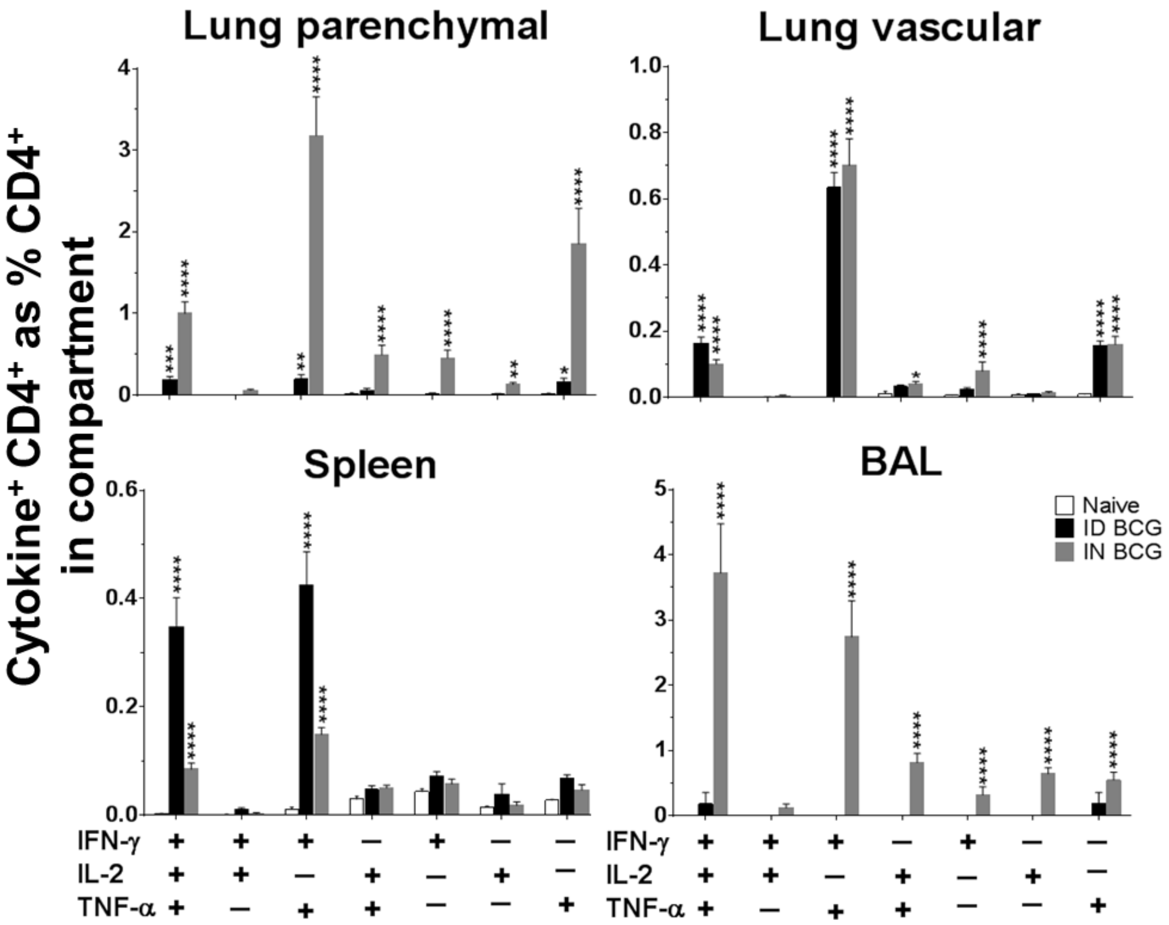

Supplementary Figure 2 Frequency of antigen-specific CD4$^+$ T cells producing each cytokine combination. Six weeks after immunisation with BCG via IN or ID route, intravascular staining & ICS identified populations of lung parenchymal and lung vascular PPD-T-specific (cytokine$^+$) CD4$^+$ T cells producing IFN-γ, TNF-α or IL-2 alone or in combination. Bars represent mean ± SEM (*n*=6). Statistical comparison is between naïve and BCG-immunised groups. Two-way ANOVA with Sidak's post-test * $P<0.05$, ** $P<0.01$, *** $P<0.001$ **** $P<0.0001$. Data representative of one of two independent experiments.
